# Deep learning-based virtual staining, segmentation, and classification in label-free photoacoustic histology of human specimens

**DOI:** 10.1038/s41377-024-01554-7

**Published:** 2024-09-02

**Authors:** Chiho Yoon, Eunwoo Park, Sampa Misra, Jin Young Kim, Jin Woo Baik, Kwang Gi Kim, Chan Kwon Jung, Chulhong Kim

**Affiliations:** 1 Departments of Electrical Engineering, Convergence IT Engineering, Mechanical Engineering, Medical Science and Engineering, Graduate School of Artificial Intelligence, and Medical Device Innovation Center, Pohang University of Science and Technology (POSTECH), Pohang, Republic of Korea; 2 Opticho Inc., Pohang, Republic of Korea; 3 Department of Health Sciences and Technology, Gachon Advanced Institute for Health Sciences and Technology (GAIHST), Gachon University, Incheon, Republic of Korea; 4 Cancer Research Institute, College of Medicine, The Catholic University of Korea, Seoul, Republic of Korea; 5 Department of Hospital Pathology, Seoul St. Mary’s Hospital, College of Medicine, The Catholic University of Korea, Seoul, Republic of Korea

**Keywords:** Photoacoustics, Microscopy

## Abstract

In pathological diagnostics, histological images highlight the oncological features of excised specimens, but they require laborious and costly staining procedures. Despite recent innovations in label-free microscopy that simplify complex staining procedures, technical limitations and inadequate histological visualization are still problems in clinical settings. Here, we demonstrate an interconnected deep learning (DL)-based framework for performing automated virtual staining, segmentation, and classification in label-free photoacoustic histology (PAH) of human specimens. The framework comprises three components: (1) an explainable contrastive unpaired translation (E-CUT) method for virtual H&E (VHE) staining, (2) an U-net architecture for feature segmentation, and (3) a DL-based stepwise feature fusion method (StepFF) for classification. The framework demonstrates promising performance at each step of its application to human liver cancers. In virtual staining, the E-CUT preserves the morphological aspects of the cell nucleus and cytoplasm, making VHE images highly similar to real H&E ones. In segmentation, various features (e.g., the cell area, number of cells, and the distance between cell nuclei) have been successfully segmented in VHE images. Finally, by using deep feature vectors from PAH, VHE, and segmented images, StepFF has achieved a 98.00% classification accuracy, compared to the 94.80% accuracy of conventional PAH classification. In particular, StepFF’s classification reached a sensitivity of 100% based on the evaluation of three pathologists, demonstrating its applicability in real clinical settings. This series of DL methods for label-free PAH has great potential as a practical clinical strategy for digital pathology.

## Abstract (web summary)

Deep learning framework for automated virtual staining, segmentation, and classification in label-free photoacoustic histology of human liver cancers.

## Graphical abstract

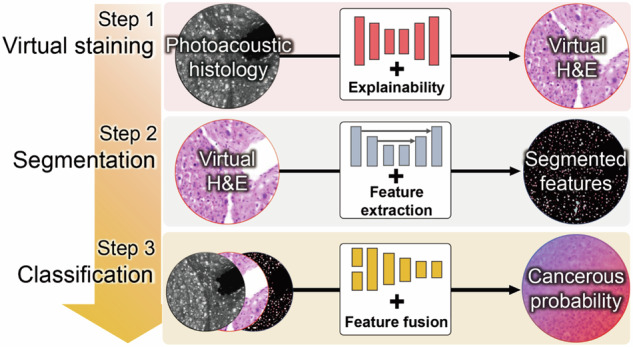

## Introduction

Histopathology, the microscopic imaging of specimens, is the primary source of diagnostic information for optimal surgical management. Histopathology and life-science research use chromatic dyes or fluorescence markers for histochemical staining to visualize tissue and cellular structures^1,2^. In particular, hematoxylin and eosin (H&E) staining is the gold standard for microscopic tissue examination in histopathology^3^. However, traditional slide preparation for staining is labor-intensive and error-prone, which presents a dilemma^4^. As the number of items requiring pathological examination increases, additional slides must be produced for staining, but an insufficient sample quantity may cause an inappropriate diagnosis.

Recently, many optical microscopic techniques have been utilized to address the issues of sample preparation and staining quality^5–9^. For example, light-sheet microscopy^10,11^ rapidly images large specimens with intrinsic optical sectioning, but it typically involves additional chemical procedures such as optical clearing and fluorescence dyeing. As label-free imaging modalities, bright-field microscopy (BF)^12^, optical coherence tomography (OCT)^13^, and autofluorescence microscopy (AF)^6,14^ provide histopathological images with a simplified sample preparation without staining. However, these methods are less able than H&E staining to identify specific biomolecules and have difficulty providing sufficient clinical information. Raman microscopy^15,16^ and spectroscopic OCT^17^ resolve unlabeled biochemical composition with spectral analysis, but have relatively weak signal sensitivity. On the other hand, novel label-free imaging methods have been proposed that can acquire selective images by targeting specific excitation wavelengths. Deep-ultraviolet microscopy (DUV)^18^, photoacoustic microscopy (PAM)^19–22^, and photoacoustic remote sensing (PARS)^23^ use endogenous contrasts to visualize individual chromophores. Among these modalities, PAM is a promising high-sensitivity imaging technology that selectively highlights biomolecules based on optical absorption^19,24–31^. In particular, DNA/RNA highly absorbs ultraviolet (UV) light, allowing UV-PAM to visualize cell nuclei without staining^32^, and thus this technique has been intensively explored as a label-free histological tool (i.e., photoacoustic histology (PAH))^33,34^. In clinical applications, however, label-free PAH techniques are still challenging to provide color-coded high-resolution histopathological images comparable to familiar H&E-stained whole slide images (WSIs). To solve this challenge, unlabeled images need to be translated into interpretable images that contain sufficient information for clinical diagnosis.

The development of deep learning (DL)-based image processing, including virtual staining and histological image analysis (HIA), has greatly expanded the clinical utilization of label-free images^35–42^. First, virtual staining allows images obtained from label-free microscopy to mimic the morphological characteristics revealed by various histochemical staining styles. However, traditional DL methods have used supervised learning algorithms that require image pairs^43–48^, which involve a difficult image registration process during data pre-processing. As an alternative, researchers have employed unsupervised image transformation methods such as cycle-consistent generative adversarial networks (CycleGAN)^49^, which are sufficient for network training even with unpaired datasets. By incorporating CycleGAN^50–55^, label-free microscopy can generate virtually stained histological images. However, when images in one domain contain more information than images in the other domain, the cycle consistency in CycleGAN can yield poor reconstruction, and using two generators and two discriminators is memory-intensive and time-consuming. Pérez et al^56^. presented a virtual staining method using contrastive unpaired translation (CUT)^57^ by maximizing the mutual information between generated and input images. The CUT model uses patch-wise contrastive learning to achieve better virtual staining quality and is lighter and faster than CycleGAN. However, for application to safety-critical medical data analysis, the black box problem, where the process of deriving conclusions by DL is unknown, requires further investigation. Secondly, DL-based HIA, another DL-based image processing method, is a crucial step in the early stages of histological image diagnosis. DL-based HIA uses an automated analysis system that enables objective evaluation and reduces the cost of diagnosis. Numerous DL-based HIA tasks have been proposed, including image classification^58,59^, object or lesion identification^60^, and nuclei segmentation^61^. However, most DL-based HIAs primarily rely on conventional histopathological images, and they often are incompatible with label-free images. Even when they do work with label-free images, they are typically designed for a single HIA task^45,50,52,62^. There is a clear need for a DL-based HIA that is compatible with label-free images and can provide sensitive and accurate analysis results.

**Fig. 1:**
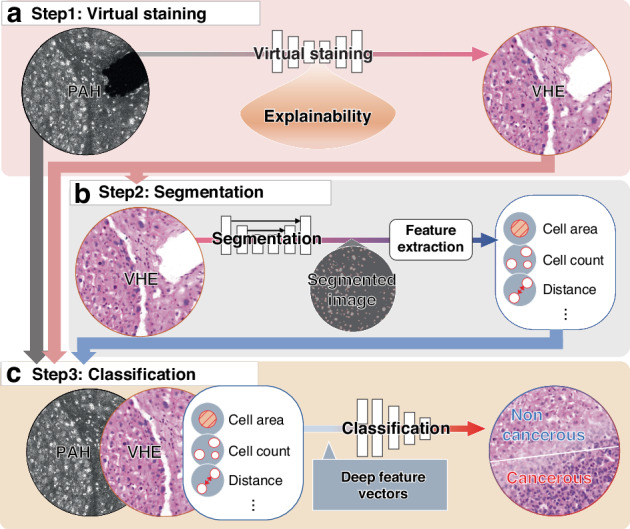
**DL-based framework for automated HIA to perform virtual staining, segmentation, and classification of label-free PAH.** **a** Virtual staining sequence with explainability to generate VHE images with label-free PAH. PAH photoacoustic histology image; and VHE virtual staining H&E. **b** Segmentation sequence to generate features: cell area, cell count, and distance. **c** Classification sequence to classify cancerous or not with PAH, VHE, and segmentation deep feature vectors

In this paper, we develop a DL-based framework for automated HIA that performs virtual staining, segmentation, and classification in label-free PAH images of liver cancer (Fig. 1). PAH images can reveal histological characteristics, but they also present pathologists with relatively less familiar images that can make diagnosis challenging. As a first step, an explainable-CUT (E-CUT) approach is proposed for virtual staining to transform grayscale PAH images to virtual H&E-stained images (Fig. 1a). For virtual staining, E-CUT uses saliency loss and integrated gradients to not only preserve image content but also visualize saliency information and feature attribution to increase traceability. Next, it performs image segmentation and feature extraction to extract information for further analysis (Fig. 1b). Features such as the cell area, cell count, and distance between cells are extracted from the segmented images. Finally, a DL-based classification model using a stepwise feature fusion method (StepFF) is proposed, using the combined PAH, VHE, and segmentation deep feature vectors (DFVs) to classify noncancerous and cancerous liver cells. The performance of StepFF is compared with that of traditional H&E (Fig. 1c). This multi-step DL-based framework not only transforms PAH images to H&E-style ones for clinical applicability but also enables accurate analysis by fusing multiple DFVs. The innovative single framework for virtual staining, segmentation, and classification unfolds several insights as follows: (1) label-free histological images are obtained using UV-PAM with high sensitivity. (2) The explainable DL-based unsupervised virtual staining technique (E-CUT) is devised, which can highlight histologically significant morphologies in the input data and input features that significantly contribute to discriminator prediction. (3) Biological features are clearly extracted from the VHE images using a U-Net-based segmentation technique. (4) The DL-based stepwise feature fusion method (StepFF) is presented by combining the deep feature vectors, which pathologically classifies human hepatocellular carcinoma (HCC) images. The superiority of the proposed system is confirmed by comparing with reported DL-based label-free histology techniques (Table S1).

## Results

### Label-free PAH system

**Fig. 2:**
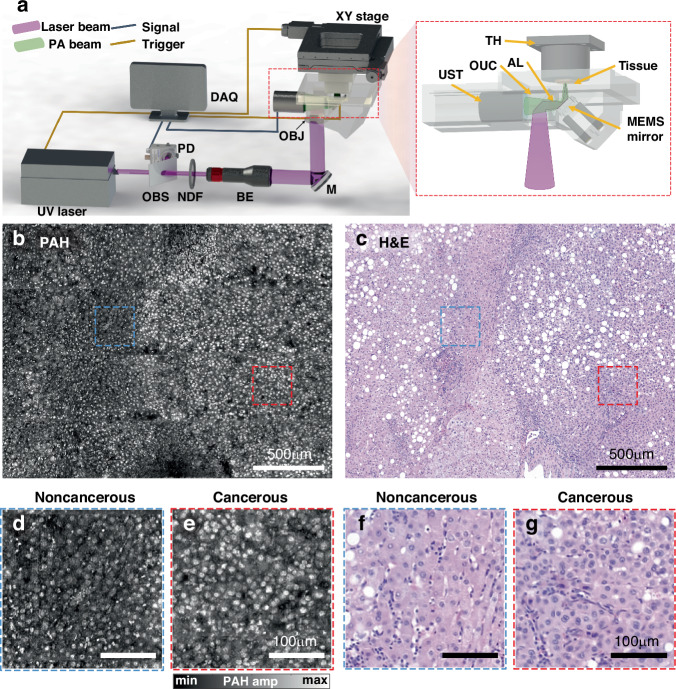
**Label-free PAH imaging system.** **a** Schematic of the PAH system and the close-up of the signal acquisition module. PD photodiode, OBS optical beam splitter, NDF neutral density filter, BE beam expander, M Mirror, OBJ objective lens, OUC opto-ultrasound beam combiner, DAQ data acquisition, UST ultrasound transducer, AL acoustic lens, and TH tissue holder. **b** PAH image of human liver tissue. **c** Corresponding H&E-stained image. Scale bars, 500 μm. H&E, hematoxylin and eosin stained image. **d**, **e** Zoomed-in PAH images of noncancerous and cancerous regions, respectively. **f**, **g** Zoomed-in H&E images of noncancerous and cancerous regions, respectively. Scale bars, 100 μm

PAH images of human liver samples were obtained using a previously developed UV-PAM system^63^. For label-free DNA/RNA-selective imaging, the PAH system employed a pulsed UV laser with a center wavelength of 266 nm and a pulse repetition rate of 20 kHz^64,65^ (Fig. 2a). The zoomed-in image in Fig. 2a is a detailed schematic of the PA signal acquisition module. A formalin-fixed paraffin-embedded (FFPE) tissue section was fixed to the tissue holder, and the laser and acoustic beams were simultaneously scanned with a MEMS mirror. After passing through an opto-ultrasound combiner with an acoustic lens, acoustic waves were collected by an ultrasound transducer with a center frequency of 20 MHz. The imaging system has a lateral resolution of ~1.2 μm^63^, and it takes ~35 s to image a field of view of 700 × 1000 μm^2^ (i.e., one piece of the PA image), with a step size of 1.0-micrometer per pixel. Figure 2b shows a PAH maximum amplitude projection (MAP) image of the human liver tissue section. A PA whole slide mosaic image was generated by stitching together 123 pieces (with a total area of 10.5 × 8.0 mm^2^), according to the scanning geometry of the motorized XY stage. The corresponding H&E WSI was obtained by imaging a slice adjacent to the slice used for the PAH image (Fig. 2c). Compared to the images from the noncancerous region (Fig. 2d), higher cell densities and larger cell nuclei can be identified in the zoomed-in PAH images acquired in the cancerous region (Fig. 2e). The PAH images are highly correlated with the traditional H&E images (Fig. 2f, g). Detailed quantitative analyses will be discussed in the following sections.

### Explainable contrastive unpaired translation (E-CUT) VHE network

**Fig. 3:**
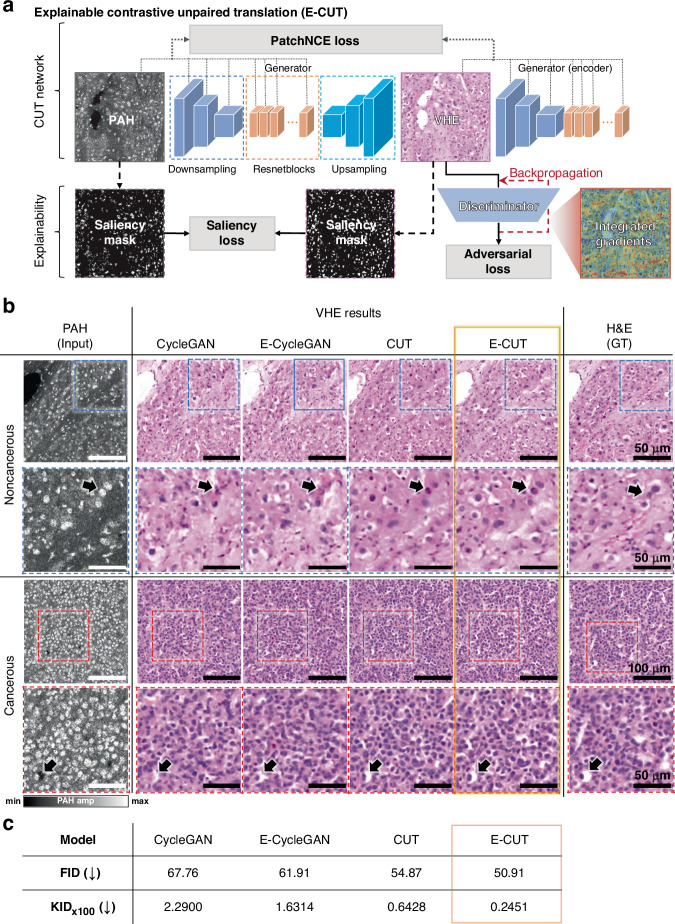
**Overall virtual staining network architecture and results.** **a** Explainable contrastive unpaired translation (E-CUT) network architecture. **b** Visual comparison of PAH (input), and VHE results with various networks: CycleGAN, explainable CycleGAN (E-CylcleGAN), CUT, E-CUT, and H&E (ground truth, GT). Scale bars, 100 μm. Zoomed-in images scale bars, 50 μm. Black arrows highlight cell nuclei, showing the difference in morphology preservation between different networks. **c** Quantitative comparison results for different VHE networks with FID and KID scores. Results are evaluated on a total of 100 test tiles. FID Fréchet Inception Distance, KID Kernel Inception Distance

After acquiring grayscale PAH images of the human liver tissue section, the proposed unsupervised DL method, E-CUT, was implemented for virtual staining. E-CUT is based on patch-wise contrastive learning and incorporates additional explainable components such as saliency loss and integrated gradients (Fig. 3a). Saliency loss continuously tracks both saliency masks from PAH and VHE to assist in resolving singularity issues that may arise during the training phase (Fig. S1). The saliency mask improves explainability by highlighting the important morphology of input data in the virtual staining process and visualizing the model’s ability to preserve structural information^66–69^. Another one of the explainable components, the integrated gradients, can highlight the most influential features and increase the explainability of the model^70^. The discriminator’s integrated gradients allow the identification of features that are important in the process of determining whether the image generated by the generator is real or fake. Note in Fig. S1 that as training progresses, the previously randomly emphasized integrated gradients gradually focus on input features around cell nuclear information, which is the information of interest in pathological virtual staining. Subsequently, the virtual staining results of E-CUT were compared with other unsupervised DL methods, such as CycleGAN^49^, explainable CycleGAN^52^ (E-CycleGAN), and CUT^57^ (Fig. 3b, c). The CycleGAN model has a cyclic (bidirectional) structure consisting of two generators and two discriminators that can learn to transform to another domain while preserving the content of the input image. However, CycleGAN is still limited in preserving detailed structural information, so E-CycleGAN incorporates additional explainable components to preserve the more precise structure and increase the explainability of the model (Fig. S2). On the other hand, CUT employs patch-wise contrastive learning, offering better virtual staining results than CycleGAN, with the added advantage of being lighter, i.e., using relatively fewer generators and discriminators. The proposed E-CUT also incorporates additional explainable components in the CUT, which enable better preservation of structural information and increase the explainability of the model.

We compared the performance of the four virtual staining models and validated their results against the original H&E images. Figure 3b shows the original PAH (input) and H&E (ground truth) images of the human liver tissue section, and the corresponding VHE results processed with the four virtual staining models for each noncancerous and cancerous case. For a detailed analysis of the staining results, each image is zoomed-in (dotted boxes). Especially in the areas indicated by the black arrows, the VHE results of CycleGAN, E-CycleGAN, CUT, and E-CUT, in that order, show better preservation of the cell nucleus morphology, with staining similar to that in the real H&E image. In particular, the structural aspects of the cell nuclear information in the input PAH images are well preserved in the E-CycleGAN and E-CUT with saliency loss, whereas the overall staining quality (e.g., degree of color and morphology reproduction) is improved in CUT and E-CUT with PatchNCE loss^57^. As a result, E-CUT demonstrates a remarkable capability to effectively follow the morphology of PAH images, thereby contributing significantly to the overall staining quality. Hematoxylin staining is more prominent following the selective visualization of cell nuclei in the PAH images. Including the eosin staining for RBCs, collagen, and smooth muscle, the proposed VHE yields diagnostic histological images. The enhanced staining quality makes the E-CUT results more closely resemble real H&E staining. However, it is important to note that the VHE does not perfectly match the morphology obtained from H&E. As discussed in the previous section, we used two adjacent slides, and the ground truth H&E image is not perfectly registered to the PAH image.

For a quantitative comparison, we applied the Fréchet Inception Distance (FID)^71^ and the Kernel Inception Distance (KID)^72^ (Fig. 3c). Both the FID and KID metrics evaluate the performance of the image generation model, calculating the difference between the generated image and the real one. The obtained lower values for both metrics indicate that the distributions of the two data are closer, which implies the staining quality is closer to the ground truth. This finding confirms that E-CUT outperforms than above-mentioned existing models in terms of FID by a large margin (~4 to 17 difference). E-CUT also achieves the lowest KID (0.2451) on the PAH to VHE translation. Notably, E-CycleGAN and E-CUT, which utilize saliency masks, exhibit superior performance to conventional CycleGAN and CUT. Similarly, CUT and E-CUT, employing contrastive learning, outperform CycleGAN and E-CycleGAN. E-CUT has greater stability and dependability because the saliency loss ensures that the extracted saliency mask of the input PAH image stays consistent when transferred to the H&E domain, thus achieving the best FID and KID scores among all virtual staining methods. Since the FID and KID scores comprehensively evaluate color, texture, and structure, these results also indicate that E-CUT is good at reproducing the original color and morphology.

### U-Net-based feature segmentation network

**Fig. 4:**
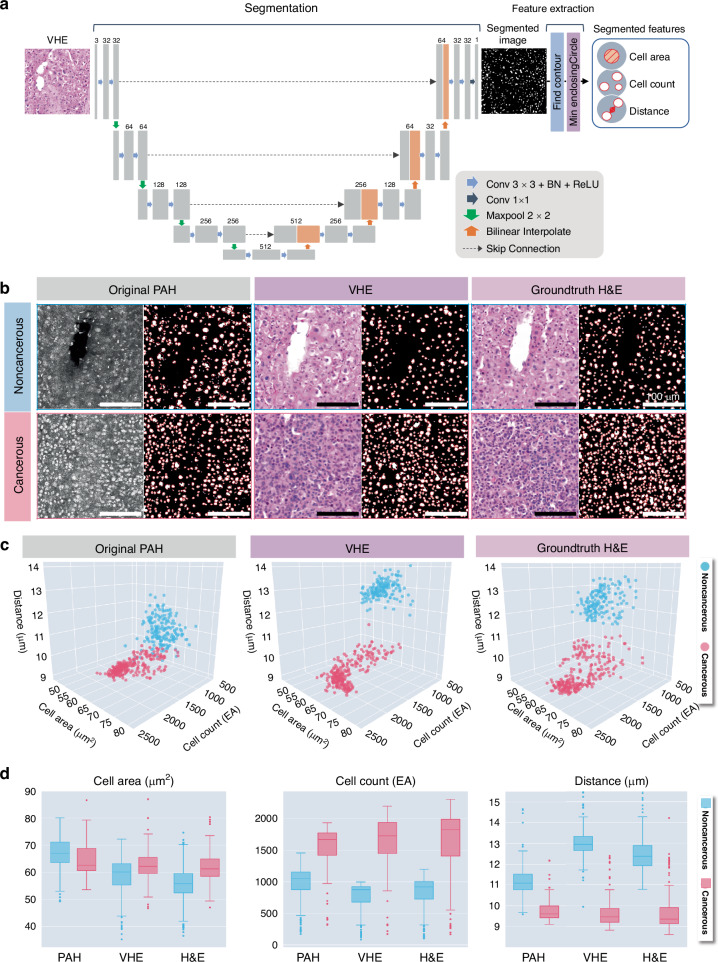
**Overall feature segmentation network architecture and results.** **a** U-Net-based feature segmentation network architecture with two phases: segmentation and feature extraction. **b** Segmentation of cell nuclei in PAH, VHE, and H&E images. Scale bars, 100 μm. **c** 3D scatter plot of each feature in the PAH, VHE, H&E images. The blue and red dots represent noncancerous and cancerous cases, respectively. **d** Cell area, cell count, and distance features are calculated from the PAH, VHE, and H&E images

The feature segmentation network, illustrated in Fig. 4a, has two primary components: a segmentation module that acquires a segmented image (i.e., cell nucleus mask information) from the input images, and a feature extraction module that extracts structural information about cell nuclei from the segmented images. The segmentation module is based on the fundamental U-Net architecture^73^, comprising a model with contraction and expansion paths, each consisting of four layers. For versatility, the segmentation model was trained and evaluated with a public H&E dataset^74–76^ and the results are presented in Table S2. This trained segmentation model was used to create cell nucleus segmented images from PAH, VHE, and H&E datasets. Afterward, in the feature extraction module, the cell segmented images were analyzed using OpenCV tools^77^ (e.g., findContours and minEnclosingCircle) to extract the cell area, cell count, and average intercellular distance. These features are clinically representative of differences between noncancerous and cancerous tissues.

Figure 4b shows examples of PAH, VHE, and H&E images and the segmented images for each. In all imaging modalities, the cell nucleus density is significantly higher in cancerous tissues than in noncancerous tissues. For more detailed analyses, the segmented features for PAH, VHE, and H&E were extracted from the segmented images (Table S3) and visualized in 3D scatter plots (Fig. 4c). We marked the averaged values for features in each test tile image. To remove outliers and visualize the correlation between major features, the interquartile range (IQR) was employed^78^. In common, the cancerous tissues (red dots) show higher cell counts, shorter intercellular distances, and higher densities than the noncancerous tissues (blue dots). However, in the PAH images, the noncancerous and cancerous features overlap considerably, making the distinction unclear. In contrast, the VHE and H&E images clearly separate these features. Figure 4d shows the detailed distribution of segmentation features (i.e., cell area, cell count, and distance). Due to its limited resolution and contrast, the PAH image has a greater cell area than the VHE and H&E images. Moreover, the trend of the features distinguishing between cancerous and noncancerous cells in the PAH image appears to be slightly reversed, which is addressed by the VHE. In the cell count and intercellular distance comparisons, both the PAH and VHE images tend to be similar to the H&E images, with a higher cell count and closer distance for the cancerous case. Based on the H&E, the PAH and VHE images show error rates of 14.51% and 6.74%, respectively (Table S3), which suggests that the VHE image has similar features to the ground truth H&E. Overall, the segmentation results imply that effective histopathological analysis is possible, because the limitations of images and features in the PAH are addressed by the VHE. However, considering the distribution without IQR (Fig. S3), it is important to note that the segmentation step alone is not enough to effectively classify the type of tissues, and additional steps are required for accurate diagnosis.

### Stepwise feature fusion classification network (StepFF)

**Fig. 5:**
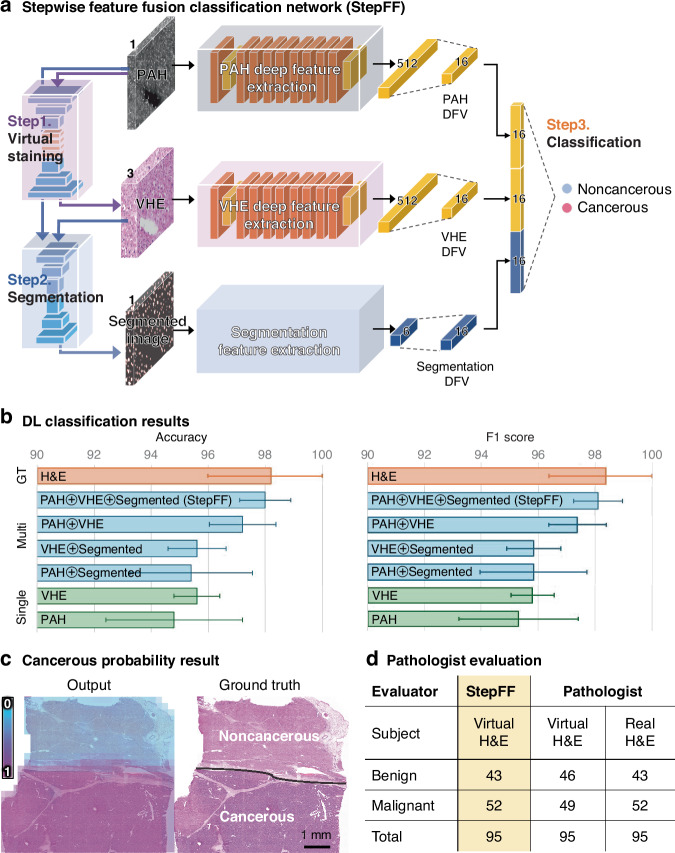
**Overall classification network architecture and results.** **a** Overall stepwise feature fusion (StepFF) classification network architecture. **b** Visualization of DL classification results for different source feature inputs. **c** Cancerous probability outputs of StepFF (0 for noncancerous, 1 for cancerous), and the ground truth. **d** Comparison of StepFF and pathologists’ classification results

Although virtual staining in label-free imaging produces VHE images that closely resemble real H&E images, image quality limitations of the source PAH image limit the ability of traditional DL-based HIA techniques to interpret features on VHE. To complement the image information and more accurately classify cancer, we propose StepFF, which integrates multiple DFVs generated in each step (Fig. 5a). Three DFVs are used for the cancer classification: PAH DFV, VHE DFV, and segmentation DFV. The deep feature extractions from the PAH and VHE images are performed using the ResNet^79^ model, which has been identified as the most suitable classification model for VHE image analysis among such well-known CNN models as EfficientNet^80^, Inception^81^, VGGNet^82^, SwinNet^83^, and ResNet^79^ (Table S4). First, in the deep feature extraction step, the PAH image is utilized to extract 512-dimensional DFV (the default dimension of the output DFV in the basic ResNet). The VHE image obtained in the virtual staining process (Step 1) is utilized to extract 512-dimensional DFV. Subsequently, in the feature segmentation (Step 2), three biological features (i.e., cell area, cell count, and distance), which are basic indicators of clinical evaluation, were extracted from PAH and VHE. Segmentation DFV were optimized by comparing classification scores according to feature combinations (Table S5). In this experiment, the best results were obtained when all the segmental features of PAH and VHE were used together, so a 6-dimensional DFV with all the segmental features of PAH and VHE is used as the segmented features for the final classification (Table S6). In the final step, all features are changed into 16-dimensional DFVs with fully connected layers to merge them fairly, then fused to classify the cancerous tissue.

Figure 5b shows a visualization of representative cross-validated DL classification results (accuracy and f1 score), and the details are in Table S6 (accuracy, f1 score, precision, and recall). Among the single-modal results, the classification performance using the VHE (accuracy of 95.60%) is better than that of the PAH (accuracy of 94.80%) as it contains more information (three-dimensional color information). The multi-modal results are better than the single-modal results because they use multiple images or additional segmentation DFV. While VHE shows little performance improvement with the segmentation DFV, PAH performs better. This difference confirms that adding segmentation DFV is effective for the less informative PAH. Also noteworthy is the significant improvement in the classification results for PAH⊕VHE (accuracy of 97.20%), a 2.4% improvement over PAH alone, and a 1.6% improvement over VHE alone. Here, combining the undistorted original data (PAH) with the generated virtual image (VHE), which has additional color information, enables more accurate classification. With a combination of PAH, VHE, and segmentation DFVs, the proposed StepFF model achieves the best classification accuracy of 98% and precision of 98.14%, which is comparable to that obtained on H&E images (98.20% accuracy and 97.24% precision).

We also checked the probability of cancer at the level of the entire WSI. The cancerous probability of each tile was color-mapped and then re-stitched to the full slide image size to visualize the cancerous probability (Fig. 5c). In this experiment, we first used all the dataset tiles (training and test) to find the probability of cancer per tile (1 for cancerous, 0 for noncancerous). We then colored each tile according to the cancer probability, 1 for purple and 0 for blue, and merged the small tiles to the original WSI size while keeping the cancer probability information. For detailed histopathological evaluation, three pathologists quantitatively graded the tile images blindly. A total of 200 test tile images (100 VHE images and 100 corresponding H&E images) were randomly shuffled and graded using the World Health Organization’s histological grading system for HCC^84^. All images distinguished malignancy clearly and morphologically differentiated HCC. The classification used two categories, benign (noncancerous) and malignant (cancerous), according to the presence of HCC. Of the 100 tiles in each of the VHE and H&E test sets, five tiles were excluded if each image had less than 20% tissue surface or only stromal cells (Table S7). The Kappa coefficient was 0.979, indicating almost perfect agreement between the pathologists’ responses^85^. Comparing the classification results between StepFF and the pathologists’ grading, StepFF showed a strong correlation with the real H&E grades (Fig. 5d). Among the 95 tiles, both gradings equally classified 43 benign and 52 malignant tiles. The results show that while classification is still difficult with VHE alone, StepFF can utilize the DFVs of multiple images and segmented features to make judgments that are nearly identical to the pathologists’ assessment based on the real H&E.

## Discussion

Although histological imaging is a routine tool for pathological diagnostics, traditional histochemical staining is laborious and error-prone. To address this, label-free imaging and DL-based HIA have been exploited to highlight oncological features according to intrinsic imaging contrasts. However, conventional single-modal techniques are still insufficient in clinical settings. In this study, we introduce a DL-based framework for automated HIA in label-free PAH. The proposed multi-modal method has three steps: virtual staining, segmentation, and classification (Note S1). First, we present a fast and accurate virtual staining method, E-CUT, that combines contrastive unpaired image transformation and explainable components. E-CUT maximizes the mutual information between the generated (VHE) images and input (PAH) images using only a single generator and discriminator. In addition, the saliency loss and integrated gradients increase explainability, providing improved similarity and traceability during the transformation between the image domains. E-CUT can learn accurate domain mappings and achieve superior performance to traditional virtual staining methods. Second, we demonstrate segmentation for morphological feature extraction. This segmentation analysis provides quantitative metrics for diagnosis from PAH, VHE, and H&E images. The segmentation in VHE shows a distinct distribution of cancerous characteristics that is very similar to that of H&E. Interestingly, in PAH, the noncancerous cell area tends to be larger than the cancerous cell area, as opposed to the VHE and H&E images. Because it was trained with public H&E^74–76^, the segmentation model does not work well with other styles, such as PAH. In the case of cancerous cells with high cell density, it is especially challenging to accurately segment all the cell nuclei. Therefore, the measured cell area in PAH images of cancer tends to be smaller than the actual cell area. For similar reasons, the cell count in cancerous PAH images is underestimated, and the intercellular distance is greater. For the final classification step, we introduce a multi-modal classification method termed StepFF, which uses PAH, VHE, and biological features together for better performance. While scarce information limits the classification performance of single-modal HIA, by integrating DFVs of each step, StepFF achieves remarkable classification performance. Notably, the StepFF’s classification results obtained after excluding the five unsuitable tiles (Table S7) show 100% sensitivity to pathologists’ evaluation, demonstrating that the StepFF performs very well in general cases.

While the proposed framework transforms label-free images to H&E-style and provides diagnostic insight, improvements are needed for better analysis. Within the virtual staining stage, the additional employment of a resolution enhancement network can provide much clearer VHE images, allowing further diagnosis by differentiating nuclear atypia in HCC. In the segmentation process, we use a segmentation model trained with public H&E datasets, which limits the segmentation performance. To improve the segmentation performance and enable further evaluation, we plan to obtain the annotation of liver PAH and H&E images. Furthermore, additional transfer learning and data augmentation can address result bias and performance limitations. In particular, the public H&E images can be used as additional training data for our proposed method by generating virtual PAH images. Finally, additional techniques for obtaining attribution maps (such as GradCAM^86^, RISE^87^, Extremal perturbations^88^, etc.) can be used to improve the explainability of the system.

In conclusion, we present a novel, explainable, interconnected DL-based framework for virtual staining, segmentation, and classification of label-free PAH images. This interconnected approach executes the three tasks simultaneously and shares outputs, resulting in improved diagnostic accuracy, time savings, and reduced sample consumption, which can be implemented in intraoperative digital pathology workflows for clinical applications. Furthermore, the multi-modal framework can be generalized across different types of cancer diagnoses and adapted to the digital histopathology of other label-free imaging modalities (e.g., AF, BF, and OCT). We expect the proposed approach to have a clinical impact as a primary histological diagnostic tool.

## Methods

### Data preparation

All histopathological procedures were conducted following regulations and guidelines approved by the Institutional Review Board of POSTECH (approval no. PIRB-2019-E013). For specimen preparation, we harvested human liver tissue with hepatocellular carcinoma, along with adjacent noncancerous tissue. The excised tissue was processed into FFPE blocks. The 10 μm-thick unstained deparaffinized FFPE tissue sections were prepared for PA imaging. PAH images were then obtained with a UV-PAM system that uses an ultraviolet (266 nm) laser for label-free imaging (Fig. 2a)^63^. Corresponding H&E-stained images were also acquired at approximately the same location as the PAH image acquisition.

For segmentation, training the model requires data with nucleus contour label information for the PAH, H&E, and VHE images. However, obtaining such annotated data is inherently challenging, primarily due to its time-consuming and labor-intensive nature. Acquiring label information for the PAH and VHE images is especially difficult because there is little pathological knowledge to guide segmenting the nucleus contours. Therefore, instead of segmenting the nucleus contour information manually, we quickly trained and tested the model using public H&E datasets containing contour information. A total of four datasets were used to train the segmentation model: CPM-15^74^, CPM-17^74^, Kumar^75^, and TNBC^76^.

### Image pre-processing and post-processing

In the pre-processing step for raw PAH images, we conducted contrast adjustment, denoising, and background erasing. For the subsequent DL training processes, WSIs of H&E slides were converted to the same size as PAH images and cropped into smaller image tiles. First, the ×20 H&E images were downsampled to match at the magnification of ×10, which is the scale of the PAH images. The PAH images were then inverted to match the background color of the downsampled H&E image (the background was set to white). For training and testing, WSIs of both H&E and PAH images were cropped into small image tiles of 512 × 512 pixels with 50% overlap. For both virtual staining and classification, these image tiles were divided into training and test sets in proportions of 5:1. On the other hand, for segmentation, we used publicly available datasets^74–76^ and cropped them to 224 × 224 pixels for training. All the tiles of PAH, H&E, and VHE images were employed as test data for the segmentation model. A fivefold cross-validation was employed to validate the segmentation and classification results. For testing, we have used altogether different tiles, which had never been used during the training phase. We also organized the test dataset to balance between cancer and non-cancer cases for classification. Additionally, since PAH and H&E images have different numbers of channels and E-CUT requires the same number of channels for the input and ground truth, 1-channel grayscale PAH images were stacked and converted to three-channel PAH images.

The final post-processing step was to stitch the small image tiles to get the original WSI. We merged them considering the 50% overlap, so that the results were summed up, and the overlapping sections were divided by the number of overlapping images.

### Explainable contrastive unpaired translation network

#### Network architectures and training

We adopted a CUT^57^ architecture for the E-CUT model to learn the unpaired image translation between label-free PAH images and corresponding histological images stained with H&E (Fig. 3a). The generator network for E-CUT, inspired by the ResNet model, consists of downsampling, residual blocks, and up-sampling parts^89^. The downsampling process encodes an input image down to the 9 residual blocks. Each residual block is designed with a skip connection where an input to the block is concatenated to an output of the block, enabling interpretation of the encoding. In the residual block, a padded convolutional layer keeps the image size constant. The residual blocks are followed by up-sampling to decode the representation to match the size of a final output image. For the discriminator network, we utilized a PatchGAN classifier^90^. This patch-level discriminator can determine whether 70 × 70 overlapping patches are real or fake, and it can be used on images of any size in a fully convolutional fashion. The final output of the discriminator is defined as the average of the classification results on all patches.

The patch information from PAH (the input) is trained to transform it into the style of the H&E (the ground truth). In particular, during the training process, our model stores the saliency mask and integrated gradients attribution map at each training step to improve explainability. To obtain the integrated gradients, we approximate the gradient integral of the discriminator model output over the input along the path to compute the importance score for each input feature. In order to obtain the attribution map, a total of 50 steps of approximation are performed using Pytorch’s captum library^91^. Finally, the trained model is used to virtually stain the test data. The training data (PAH and H&E) and virtually stained data (VHE) are reserved for use in later stages (segmentation and classification).

#### Loss function

To ensure a reliable image translation between PAH images (the source, *X*) and H&E images (the target, *Y*), it is important to define a loss function. The goal of virtual staining is to transform the input data into the target’s style, color, and shape. However, at the same time, details such as information about cell nuclei should be preserved. Therefore, as the final training loss, we used an equal combination of the adversarial, PatchNCE, and saliency losses (Fig. 3a).

Adversarial loss (${{\rm{l}}}_{{adv}}$) minimizes the differences between the output of each network and the target domain image^92^. Contrastive learning with PatchNCE loss ensures that learning proceeds in a way that maximizes the mutual information between the input and output image patches^57^, which are obtained by passing the input and output images through a generator encoder. PatchNCE loss is calculated as the average of ${{\rm{l}}}_{{PatchNCE}}(X)$ on images from domain $X$ and ${{\rm{l}}}_{{PatchNCE}}(Y)$ on images from domain $Y$, where ${{\rm{l}}}_{{PatchNCE}}(X)$ ensures that the input-output patches correspond, and ${{\rm{l}}}_{{PatchNCE}}(Y)$ serves to further prevent the generator from making unnecessary changes.

The saliency loss (${{\rm{l}}}_{{Saliency}}(X,Y)$) is the L1 loss between ${{\rm{X}}}_{{saliency}}$ and ${{\rm{Y}}}_{{saliency}}$, and it is employed to preserve similar saliency masks during transformation and to improve explainability by visualizing the saliency mask^52^. In addition to adversarial and PatchNCE losses, saliency loss is used to extract the saliency mask of the input and the generated output to check whether the structural information is well preserved during training and leads to more accurate results. The saliency losses for the source and target domains are obtained by these equations:

1 $${X}_{{saliency}}=1-{sigmoid}\left(\left(X-{X}_{{threshold}}\right)* 100\right)$$

2 $${Y}_{{saliency}}=1-{sigmoid}\left(\left(Y-{Y}_{{threshold}}\right)* 100\right)$$

The detailed process of obtaining a saliency mask can be seen in Fig. S4. To consider only the saliency information, we first convert *Y* with RGB information to grayscale data by averaging the three-channel information, then apply sigmoid to both *X* and *Y* to get the saliency information. In the last step, the image is inversed to make the saliency information equal to 1. The optimal thresholds were obtained manually through experimentation, with 90 as the *X*_*threshold*_, which yields a good saliency mask for both noncancerous and cancerous cases of PAH, and 170 as the *Y*_*threshold*_, which works well for both noncancerous and cancerous cases of VHE (Fig. S5).

Finally, the entire loss function was formulated as

3 $${{l}_{E-{CUT}}=l}_{{adv}}\left(X,Y\right)+\frac{{l}_{{PatchNCE}}\left(X\right)+{l}_{{PatchNCE}}\left(Y\right)}{2}+{l}_{{Saliency}}(X,Y)$$

#### Parameter setting and evaluation metrics

The Adam^93^ optimizer, with b1 = 0.5 and b2 = 0.999, was used to optimize the E-CUT network parameters. The model was trained for 400 epochs, with an initial learning rate of 0.0002 for the first 200 epochs and a linear decay to a zero learning rate for the next 200 epochs, with 1 mini-batch setting. During the training phase, we augmented the data with horizontal flips. In terms of time consumption, E-CycleGAN took 73,800 s for the train and 60 s for the test, while E-CUT took relatively less time, ~62,400 s for the train and 33 s for the test. The overall test time of our proposed virtual staining (E-CUT) is much faster than the staining time of a human expert (20–30 minutes).

For a fair comparison, the same configurations were used for other virtual staining models, i.e., CycleGAN, E-CycleGAN, and CUT. We used FID and KID to evaluate the virtual staining quality of the unpaired resultant image tiles. Lower values for both metrics indicate that the distributions of the two data are closer, indicating better virtual staining quality.

### U-Net-based feature segmentation network

To numerically represent the characteristics of the VHE, we segment the cell nuclei information through feature segmentation and present it as three features: cell area, cell count, and mean intercellular distance.

#### Network architectures and training

This study employs the most well-known segmentation model, U-Net, which consists of contraction and expansion paths^73^ (Fig. 4a). While expansion paths have several deconvolution layers to upsample data and produce pixel-wise segmentation, contraction paths use convolution layers to produce high-level features in downsampling. Additionally, skip connections restore the spatial information lost during the downsampling. For feature segmentation, we employed four downscaling and four upscaling layers.

In the training phase, we trained the model to segment cell nuclei information using a public dataset, and in the testing phase, we used the trained model to segment the cell nuclei information of PAH, VHE, and H&E images. Finally, the segmented cell nuclei information was analyzed using the OpenCV tool^77^ (findContours, minEnclosingCircle) to obtain the cell area, cell count, and mean distance between cell nuclei for each tile. The cell area is the average value of the cell nuclei size, the cell count is the number of cell nuclei, and the mean intercellular distance is the average distance between cell nuclei.

#### Parameter setting and evaluation metrics

The U-Net-based segmentation model used Adam optimizer with b1 = 0.9 and b2 = 0.999, a learning rate of 0.0001, and a mini-batch size of 64, and was trained for 300 epochs. We used a combination of binary cross entropy and dice losses and allowed the training to stop early, depending on the validation loss^94^. We also used horizontal and vertical flips in the training phase to augment the data. In the last test phase, test time augmentation (TTA) was applied to get more accurate segmentation information. To visualize the results, we used the data visualization package Plotly^95^ to plot 3D spatial scatter plots and box plots (Fig. 4c, d). The total training time for the 5-fold cross-validation of feature segmentation took ~1300 s, and the test took a total of 165 s.

### Stepwise feature fusion classification network

#### Network architectures and training

In applying our StepFF method, we used the ResNet-18 model^79^ as the base model for classification (Fig. 5a). The 1-channel PAH image and the 3-channel VHE image were processed separately through ResNet-18 and processed into a fully connected layer to obtain 16-dimensional DFV from each. After that, to generate segmentation DFV from the previous feature segmentation results, three features (cell area, cell count, and distance) each from PAH and VHE were normalized with the mean and standard deviation of each segmented feature. Then, the same 16-dimensional DFV was generated using a fully connected layer with the normalized three biological features. Finally, we concatenated the three types of 16-dimensional DFVs into a 48-dimensional DFV and passed them through the last fully connected layer to classify the final non-cancer and cancer information. To compare the DL classification results with different source feature inputs (Table S6), we used a single ResNet for a single modality image (e.g., H&E, PAH, and VHE), and two ResNets for multiple modality images (e.g., PAH⊕VHE). After using ResNet to obtain DFVs, we used fully connected layers in the same way as StepFF to obtain the final cancer classification results.

#### Parameter setting and evaluation metrics

For the classification, we used the Adam optimizer to train the model for 1000 epochs, with b1 = 0.9 and b2 = 0.999, with a learning rate of 0.0001 and a mini-batch size of 32. We used focal loss for the imbalance data and allowed the training to terminate early based on the validation loss. We also used horizontal and vertical flips in the training phase to augment the data. The classification results were evaluated in terms of their accuracy, F1 score, precision, and recall. The fivefold cross-validation for StepFF took a total of 6000 s of training time and 16 s of testing time.

For further analysis, we combined the results from each tile to create one large WSI cancerous probability map, which showed the overall cancerous classification results (Fig. 5c). For this cancerous probability map, both training and test data tiles were used, in the following order. First, for each tile, StepFF’s cancerous prediction result was represented as a value between 0 and 1 and color-mapped (1 to purple, 0 to blue). Then, each tile was combined and reconstructed into the original WSI by using the post-processing method introduced in the image pre-processing & post-processing part.

### Pathologists’ evaluation

To compare StepFF’s results with clinical diagnoses, we compared them with three pathologists’ evaluation (Fig. 5d). We randomly shuffled 100 virtual staining results from StepFF together with a second group of 100 H&E images, then presented them all to three pathologists for evaluation at the same time. The pathologists were asked to determine whether each tile was noncancerous or cancerous according to the World Health Organization’s histological grading system for HCC. Five tiles with tissue coverages of 20% or less, which made the determination difficult, were excluded from the evaluation (Table S7). To measure the inter-pathologist agreement, we measured the kappa coefficient^85^, which is -1 for complete disagreement and 1 for complete agreement.

### Implementation details

The image pre-processing steps were implemented in MATLAB using R2021a (The MathWorks Inc.). All the virtual staining, segmentation, and classification sequences were implemented using Python, version 3.8.12, and Pytorch, version 1.11.0. We implemented this training and testing on a Linux system with one Nvidia GeForce RTX 3090 GPU, an AMD EPYC 7302 CPU, and 346GB of RAM.

### Supplementary information

Supplementary information

## Data Availability

The data that support the findings of this study are available on request from first author C.Y. and corresponding author, C.K. The data are not publicly available because it contains information that may violate the privacy of study participants. Supplementary information accompanies the manuscript on the Light: Science & Applications website (http://www.nature.com/lsa).

## Code availability

The code is available at https://github.com/YoonChiHo/DL-based-framework-for-automated-HIA-of-label-free-PAH-images.
