# Supplementary material for: Deep learning-based virtual staining, segmentation, and classification in label-free photoacoustic histology of human specimens
Source: Light Sci Appl. 2024 Sep 2;13:226. doi: 10.1038/s41377-024-01554-7 (PMC11369251; doi:10.1038/s41377-024-01554-7)
Supplement: Supplementary file 1 — Supplementary information [file 41377_2024_1554_MOESM1_ESM.pdf]

Supplemental Information for

Deep learning-based virtual staining, segmentation, and classification in label-free photoacoustic histology of human specimens

Chiho Yoon[1], Eunwoo Park[1], Sampa Misra[1], Jin Young Kim[1], Jin Woo Baik[1], Kwang Gi Kim[2], Chan Kwon Jung[3,4,*], and Chulhong Kim[1,*]

[1]Departments of Electrical Engineering, Convergence IT Engineering, Mechanical Engineering, Medical Science and Engineering, Graduate School of Artificial Intelligence, and Medical Device Innovation Center, Pohang University of Science and Technology (POSTECH), Pohang, Republic of Korea

[2]Department of Health Sciences and Technology, Gachon Advanced Institute for Health Sciences and Technology (GAIHST), Gachon University, Incheon, Republic of Korea

[3]Cancer Research Institute, College of Medicine, The Catholic University of Korea, Seoul, Republic of Korea

[4]Department of Hospital Pathology, Seoul St. Mary's Hospital, College of Medicine, The Catholic University of Korea, Seoul, Republic of Korea

These authors contributed equally: Chiho Yoon, Eunwoo Park, Sampa Misra

[*]Corresponding author: Chan Kwon Jung (ckjung@catholic.ac.kr)

[*]Corresponding author: Chulhong Kim (chulhong@postech.edu)

Supplementary Figures

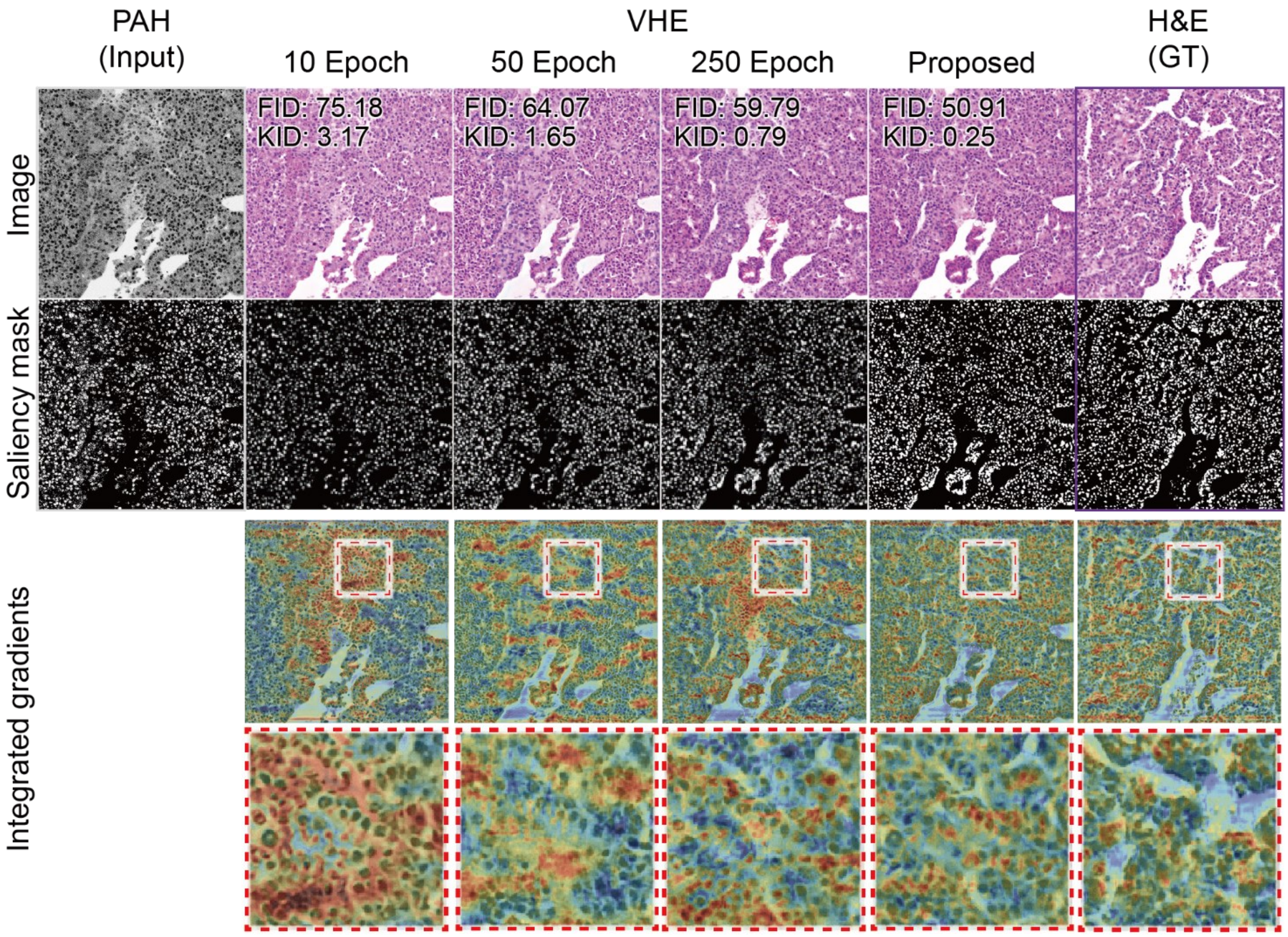

Fig. S1. Variation of virtual staining results and explainable components (saliency mask and integrated gradients) at each model training epoch. PAH, photoacoustic histology image; VHE, virtual stained H&E image; FID, Fréchet Inception Distance; and KID, Kernel Inception Distance.

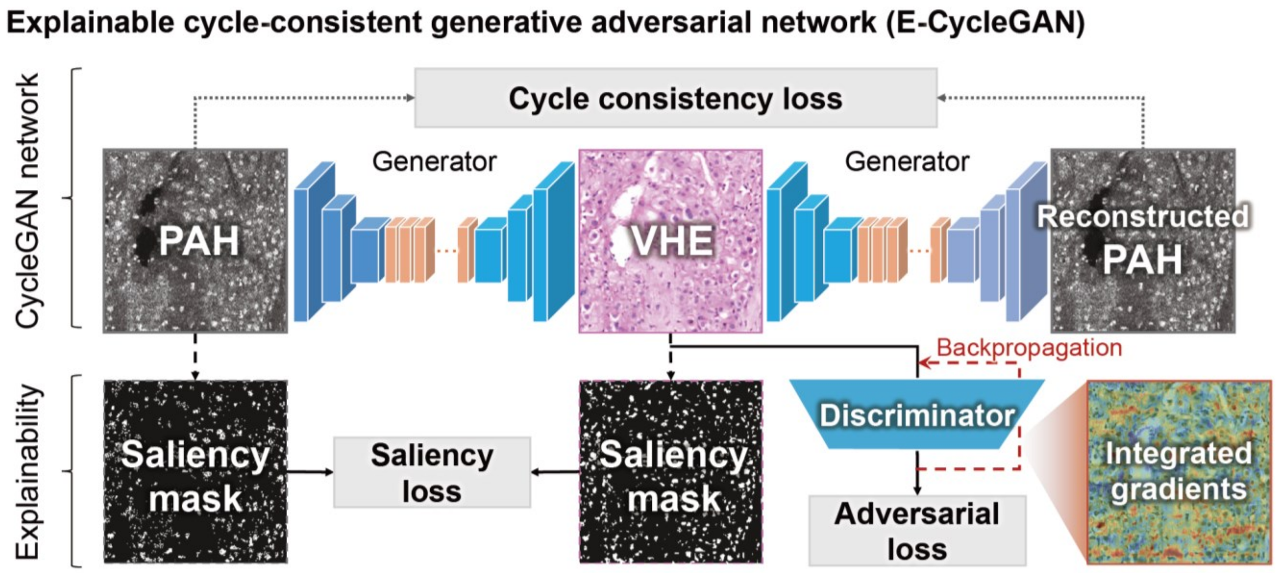

Fig. S2. Explainable cycle-consistent generative adversarial network (E-CycleGAN) network architecture. E-CycleGAN uses saliency loss in addition to the cycle consistency loss to prevent image content distortion. In addition to the saliency mask to ensure that the structural information of the cell nucleus is preserved, the integrated gradients is used to visualize the inner workings of the discriminator model to increase its explainability.

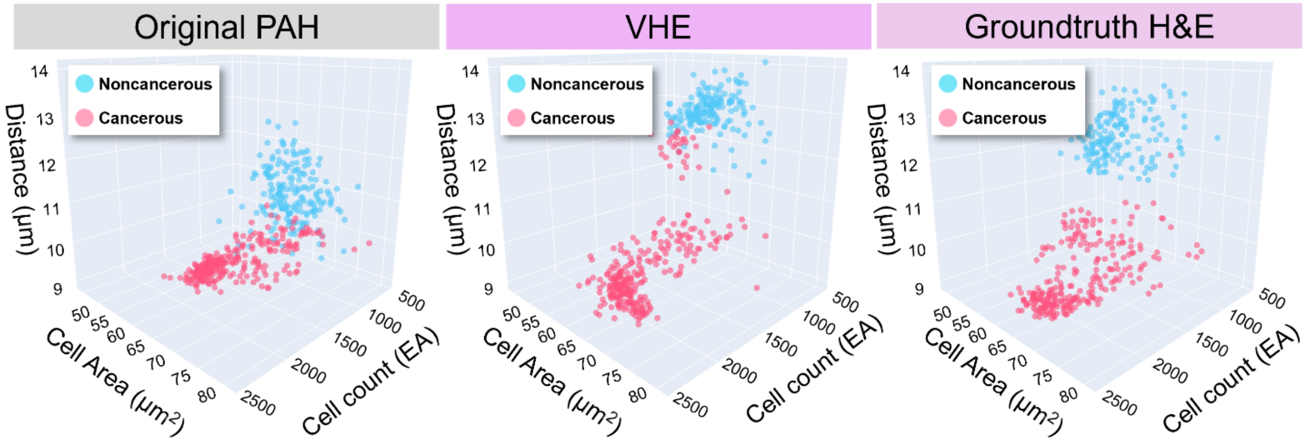

Fig. S3. 3D spatial distribution of each feature in the PAH, VHE, and H&E images without IQR. The red dots represent cancerous cases and the blue dots represent noncancerous ones.

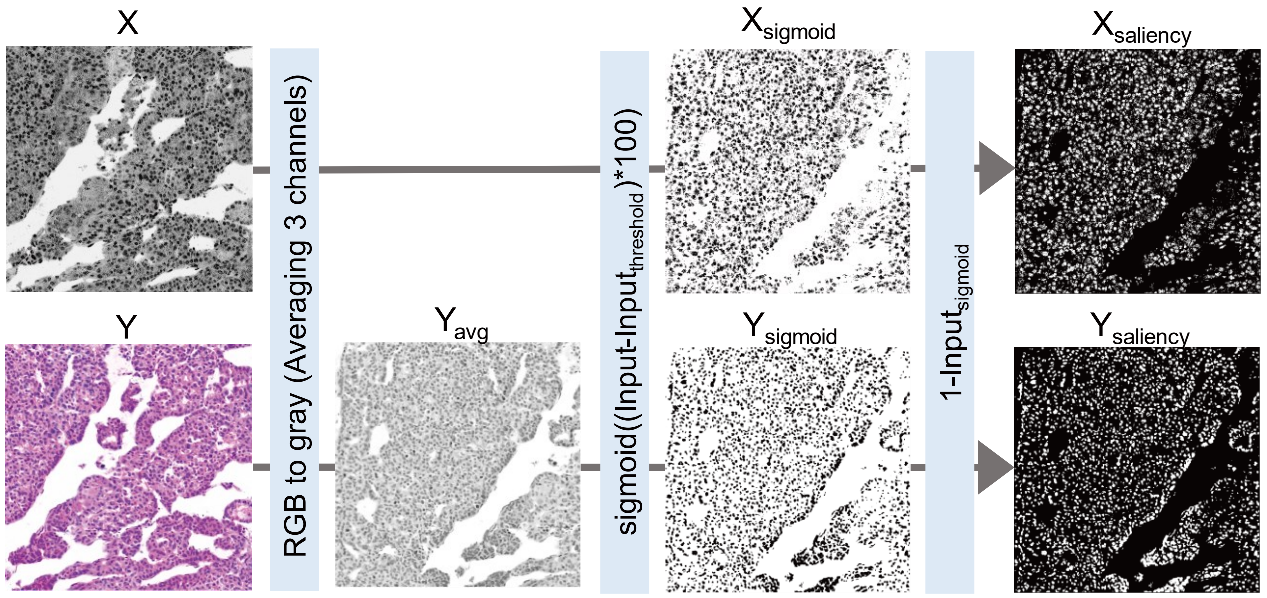

Fig. S4. Salience mask extraction sequence. X is the inverted PAH input for use in virtual staining and Y is the output VHE.

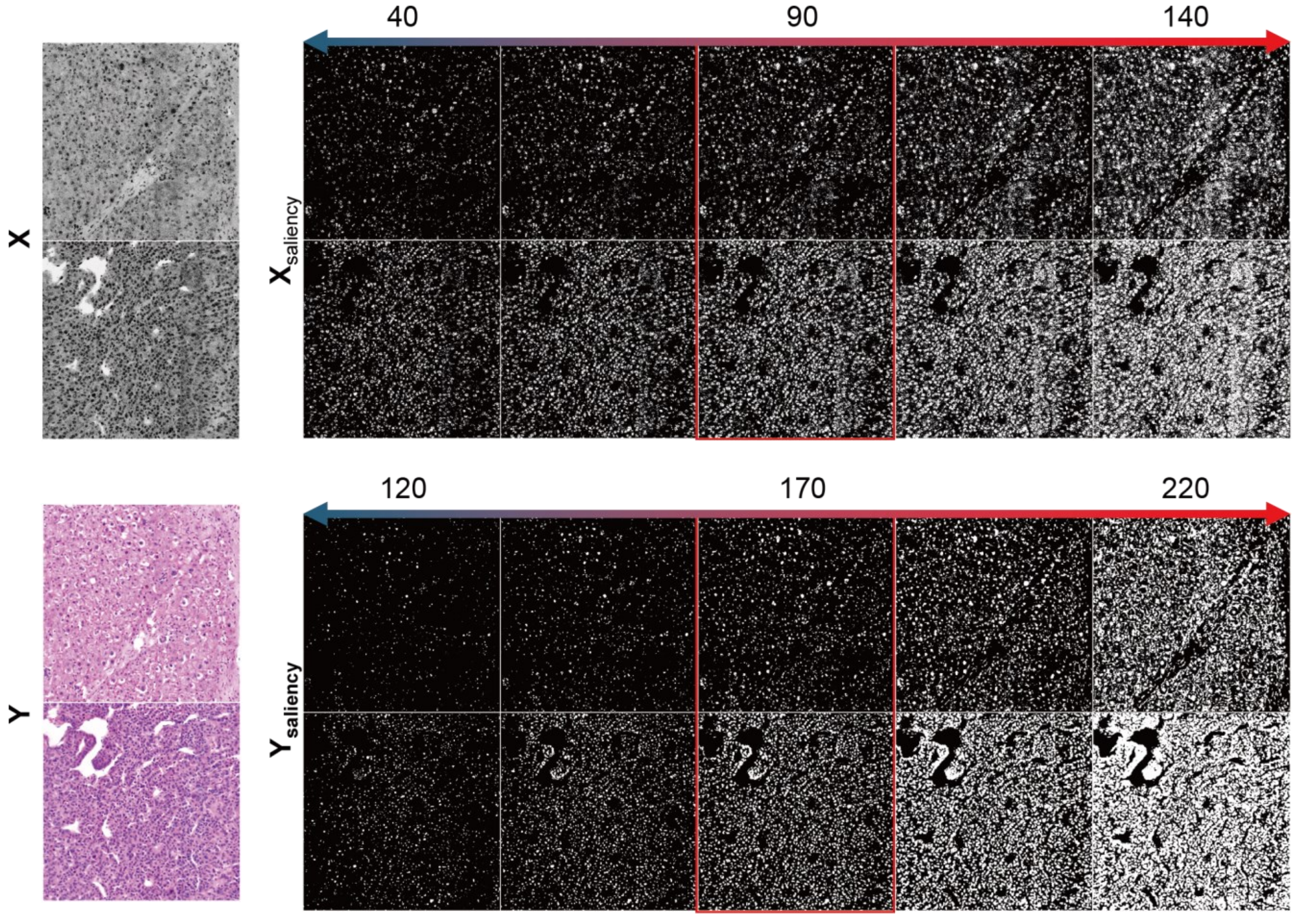

Fig. S5. Saliency mask comparison based on different threshold values. Highlighted in red boxes are the saliency masks for X (inverted PAH input) with a threshold of 90 and Y (VHE output) with a threshold of 170 used in our E-CUT model.

Supplementary Tables

Table S1. Comparison of various label-free histological techniques for visualizing human specimens with deep learning. SOCT, spectroscopic optical coherence tomography; AF, autofluorescence; BF, bright field; PARS, photoacoustic remote sensing; DUV, deep-ultraviolet; UV-PAM, ultraviolet photoacoustic microscopy; VS, virtual staining; Seg, segmentation; and Cls, classification.

References	Imaging modality	Human specimen	Unsupervised	Virtual staining	Segmentation	Classification	Explainability	Framework
Winetraub et al[1] (2021)	OCT	Skin	X	O	X	X	X	-
Tsai et al[2] (2022)	OCT	Skin	O	O	O	X	X	Integrated VS/Seg (Utilizing segmentation for virtual staining)
Rivenson et al[3] (2019)	AF	Salivary gland, Thyroid, Kidney, Liver, Lung	X	O	X	X	X	-
Picon et al[4] (2021)	AF	Colon, Breast, Lung	X	O	X	O	X	Utilizing classification for virtual staining evaluation
Meng et al[5] (2021)	AF	Ovarian	O	O	X	X	X	-
Li et al[6] (2021)	AF	Colorectal	O	O	O	X	O	Utilizing segmentation for virtual staining evaluation
Li et al[7] (2024)	AF	Autopsy	X	O	X	X	X	-
Zhang et al[8] (2022)	BF	Carotid atheroma	X	O	X	X	X	-
Kaza et al[9] (2022)	DUV	Blood smears	X	O	O	O	X	Virtual staining is utilized independently of both segmentation and classification
Liu et al[10] (2024)	Raman	Brain, Glioma	O	O	X	X	X	-
Boktor et al[11] (2022)	PARS	Skin	X	O	X	X	X	-
Martell et al[12] (2023)	PARS	Breast, Prostate	O	O	X	X	X	-
Cao et al[13] (2023)	UV-PAM	Bone	O	O	X	X	X	-
Ours	UV-PAM	Liver	O	O	O	O	O	Interconnected VS/Seg/Cls (The three tasks sharing information to enhance their performance)

Table S2. The performance of segmentation model using public dataset.

Fold	Dice (%)	F1 (%)	Precision (%)	Recall (%)
1	81.43	81.43	83.55	79.97
2	81.94	81.94	80.29	84.49
3	81.47	81.47	77.78	86.64
4	81.25	81.25	82.42	81.22
5	82.13	82.13	78.79	86.67
Mean	81.64	81.64	80.57	83.80

Table S3. Comparison of segmentation feature values and percentage error values for PAH, VHE, and H&E inputs. The difference between the ground truth H&E and the comparable was calculated using the percentage error.

		Cell area		Cell count		Distance	
Input	Type	Value (μm^2)	Per. Error (%)	Value (EA)	Per. Error (%)	Value (μm)	Per. Error (%)
Original PAH	Noncancerous	66.86±5.78	19.80	975.87±269.31	19.45	11.19±0.75	11.72
	Cancerous	64.60±5.32	2.95	1576.58±296.53	9.58	9.72±0.45	3.62
	Average	-	11.37	-	14.51	-	7.67
VHE	Noncancerous	58.85±6.95	5.45	753.21±256.20	7.80	13.24±1.17	4.43
	Cancerous	61.53±5.33	1.93	1644.51±445.79	5.68	9.70±0.90	3.44
	Average	-	3.69	-	6.74	-	3.93
Ground truth H&E	Noncancerous	55.80±7.28	-	816.95±278.94	-	12.68±1.32	-
	Cancerous	62.75±5.47	-	1743.66±429.82	-	9.38±0.54	-

Table S4. Comparison of classification performances of well-known CNN models applied to PAH. The performance of the ResNet model is superior among the various CNN models because of its extraordinarily effective feature representation and faster inference speed. The best results are shown in the highlighted line. ACC, accuracy and F1, f1 score.

Using Network	Score			
	Acc (%)	F1 (%)	Precision (%)	Recall (%)
EfficientNet	90.00 ±2.19	90.31 ±2.05	93.11 ±4.16	87.92 ±3.70
Inception	92.40 ±0.80	92.57 ±1.03	95.80 ±2.91	89.81 ±4.24
VGGNet	95.20 ±0.75	95.33 ±0.84	98.09 ±1.69	92.83 ±3.02
SwinNet	95.60 ±0.80	95.80 ±0.76	96.92 ±0.93	94.72 ±0.75
ResNet	95.60 ±0.80	95.80 ±0.76	97.05 ±2.70	94.72 ±2.50

Table S5. Comparison of scores with and without PAH and VHE segmented features. The first line is the classification result obtained by simply using PAH and VHE images together without using segmented features, and the last line is the classification result obtained by using all segmented features with PAH and VHE images. This comparison is focused on cell area, which has low discrimination as shown in Fig. 4d.

PAH segmented features			VHE segmented features			Score			
Cell count	Cell area	Distance	Cell count	Cell area	Distance	Acc (%)	F1 (%)	Precision (%)	Recall (%)
X	X	X	X	X	X	97.20±1.17	97.38±1.02	97.14±2.97	97.74±1.85
O	X	O	X	X	X	97.60±0.80	97.76±0.70	97.46±2.36	98.11±1.19
O	O	O	X	X	X	97.60±2.33	97.80±2.09	96.44±3.39	99.25±0.92
X	X	X	O	X	O	97.40±0.70	97.51±0.82	97.51±1.36	96.60±2.77
X	X	X	O	O	O	97.40±0.80	97.55±0.75	97.75±1.40	97.36±0.92
O	X	O	O	X	O	97.20±0.75	97.37±0.68	97.03±1.43	97.74±0.75
O	X	O	O	O	O	97.00±1.26	97.17±1.18	97.44±2.40	96.98±2.26
O	O	O	O	X	O	96.20±3.66	96.60±3.06	95.43±6.35	98.11±1.69
O	O	O	O	O	O	98.00±0.89	98.11±0.86	98.14±1.17	98.11±2.07

Table S6. DL classification results for different source feature inputs. The highlighted line in the table shows the performance of the proposed StepFF method with high scores close to those of H&E (GT). Paired t-tests are used to assess statistical significance across different metrics, and the null hypothesis of equality are assumed to compare the performance of the StepFF method with other models. Results are presented as (+: p-value $\leq$ 0.05) for significant difference and (≈: p-value > 0.05) for equality. ACC, accuracy; F1, f1 score; H&E, hematoxylin and eosin stained image; PAH, photoacoustic histology image; and VHE, virtual H&E image.

**Modality**	**Source feature**	**Score**			
		**Acc (%)**	**F1 (%)**	**Precision (%)**	**Recall (%)**
Single modal	H&E (GT)	98.20±2.23	98.37±1.99	97.24±3.96	99.62±0.75
	PAH	94.80±2.40 (+)	95.31±2.10 (+)	92.47±4.49 (+)	98.49±1.41 (≈)
	VHE	95.60±0.80 (+)	95.80±0.76 (+)	97.05±2.70 (≈)	94.72±2.50 (+)
Multi modal	PAH⊕Segmented	95.40±2.15 (+)	95.83±1.88 (+)	93.08±3.76 (+)	98.87±1.51 (≈)
	VHE⊕Segmented	95.60±1.02 (+)	95.84±0.95 (+)	96.25±2.03 (+)	95.47±1.51 (+)
	PAH⊕VHE	97.20±1.17 (+)	97.38±1.02 (+)	97.14±2.97 (≈)	97.74±1.85 (≈)
	PAH⊕VHE⊕Segmented (StepFF)	98.00±0.89	98.11±0.86	98.14±1.17	98.11±2.07

Table S7. Comparison of the results between the proposed StepFF and GT with the exclusion reasons for the cases excluded from the pathologist evaluation. The highlighted lines are cases where the StepFF and GT show different decisions.

**Excluded Cases**	**StepFF**	**GT**	**Pathologists comment**
1	Benign	Benign	Stromal cells only
2	Benign	Benign	Stromal cells only
3	Benign	Benign	Tissue amount under 20%
4	Malignant	Benign	Tissue amount under 10%
5	Benign	Malignant	Tissue amount under 20%

Supplementary Information

Note S1: Selection criteria for the DL models

The proposed framework consists of virtual staining, segmentation, and classification models. E-CUT and StepFF are proposed for virtual staining and classification. The widely used U-Net is used for segmentation.

Virtual staining:

• We have compared the proposed E-CUT method with the commonly used method (CycleGAN) for virtual staining in terms of performance and time complexity.

• E-CUT preserves the morphological aspects of the cell nucleus and cytoplasm, making VHE images highly similar to real H&E ones

Segmentation:

• U-Net can train with very few annotated images and can do image segmentation without requiring numerous runs.

• Without the need for time-consuming manual annotation, U-Net can automatically extract important features and perform precise segmentation by training on a sizable labeled dataset.

• An encoding path (contracting path) and a decoding path (expanding path) are features of the network's distinctive architecture that enable it to gather both local and global context. By bridging the gap between the encoding and decoding paths, skip connections enable more accurate segmentation while preserving important information from earlier layers.

• During training, U-Net uses data augmentation and regularization approaches to strengthen its robustness and generalization capabilities.

Classification:

• A deep residual network (ResNet) is used for image classification to take advantage of its low training error.

• The classification accuracy is improved by faster training because the available shortcut connections protect the network from the vanishing gradient problem and avoid negative outcomes while increasing network depth.

References

1. Winetraub, Y. et al. OCT2Hist: non-invasive virtual biopsy using optical coherence tomography. *medRxiv*. http://dx.doi.org/10.1101/2021.03.31.21254733 (2021).
2. Tsai, S. T. et al. H&E-like staining of OCT images of human skin via generative adversarial network. *Applied Physics Letters* **121**, 134102 (2022).
3. Rivenson, Y. et al. Virtual histological staining of unlabelled tissue-autofluorescence images via deep learning. *Nature Biomedical Engineering* **3**, 466-477 (2019).
4. Picon, A. et al. Autofluorescence image reconstruction and virtual staining for in-vivo optical biopsying. *IEEE Access* **9**, 32081-32093 (2021).
5. Meng, X. Y., Li, X. & Wang, X. A computationally virtual histological staining method to ovarian cancer tissue by deep generative adversarial networks. *Computational and Mathematical Methods in Medicine* **2021**, 4244157 (2021).
6. Li, X. Y. et al. Unsupervised content-preserving transformation for optical microscopy. *Light*: *Science & Applications* **10**, 44 (2021).
7. Li, Y. Z. et al. Virtual histological staining of unlabeled autopsy tissue. *Nature Communications* **15**, 1684 (2024).
8. Zhang, G. H. et al. Image-to-images translation for multiple virtual histological staining of unlabeled human carotid atherosclerotic tissue. *Molecular Imaging and Biology* **24**, 31-41 (2022).
9. Kaza, N., Ojaghi, A. & Robles, F. E. Virtual staining, segmentation, and classification of blood smears for label-free hematology analysis. *BME Frontiers* **2022**, 9853606 (2022).
10. Liu, Z. J. et al. Virtual formalin-fixed and paraffin-embedded staining of fresh brain tissue via stimulated Raman CycleGAN model. *Science Advances* **10**, eadn3426 (2024).
11. Boktor, M. et al. Virtual histological staining of label-free total absorption photoacoustic remote sensing (TA-PARS). *Scientific Reports* **12**, 10296 (2022).
12. Martell, M. T. et al. Deep learning-enabled realistic virtual histology with ultraviolet photoacoustic remote sensing microscopy. *Nature Communications* **14**, 5967 (2023).
13. Cao, R. et al. Label-free intraoperative histology of bone tissue via deep-learning-assisted ultraviolet photoacoustic microscopy. *Nature Biomedical Engineering* **7**, 124-134 (2023).
